# Nitrogen availability shapes evolution of phage resistance in cyanobacteria

**DOI:** 10.1093/ismejo/wraf180

**Published:** 2025-08-18

**Authors:** Maimona Higazi, Dikla Kolan, Esther Cattan-Tsaushu, Zohar Freiman, Shira Ninio, Sarit Avrani

**Affiliations:** Department of Evolutionary and Environmental Biology, University of Haifa, Haifa 3498838, Israel; The Institute of Evolution, University of Haifa, Haifa 3498838, Israel; Department of Evolutionary and Environmental Biology, University of Haifa, Haifa 3498838, Israel; The Institute of Evolution, University of Haifa, Haifa 3498838, Israel; Department of Evolutionary and Environmental Biology, University of Haifa, Haifa 3498838, Israel; The Institute of Evolution, University of Haifa, Haifa 3498838, Israel; Kinneret Limnological Laboratory (KLL) Israel Oceanographic and Limnological Research (IOLR), Migdal 1495000, Israel; The Institute of Evolution, University of Haifa, Haifa 3498838, Israel; Kinneret Limnological Laboratory (KLL) Israel Oceanographic and Limnological Research (IOLR), Migdal 1495000, Israel; Department of Evolutionary and Environmental Biology, University of Haifa, Haifa 3498838, Israel; The Institute of Evolution, University of Haifa, Haifa 3498838, Israel

**Keywords:** cyanobacteria, nitrogen fixation, phage, resistance, cost of resistance, tradeoff, genome evolution, glycosyltransferase, heterocyst

## Abstract

Nitrogen-fixing cyanobacteria play a key role in nitrogen and carbon biogeochemical cycles in aquatic ecosystems. Under nitrogen-limited conditions, their ability to fix nitrogen provides an advantage over other species and enables them to form harmful blooms, which are increasing in frequency and negatively impact aquatic environments. Cyanophages (viruses infecting cyanobacteria) impose strong selective pressures on these populations, and although cyanobacteria can rapidly evolve resistance to these phages, there is a tradeoff between phage resistance and nitrogen fixation. Therefore, it remains unclear whether nitrogen-fixing cyanobacteria can evolve resistance without compromising nitrogen fixation under bloom-inducing nitrogen starvation. Here, we explore the evolution of phage resistance in nitrogen-fixing cyanobacteria (*Nostoc* sp. strain PCC 7120 and *Cylindrospermopsis raciborskii*) under nitrogen starvation. We found that phage-resistant strains evolved under nitrogen starvation, although resistance emerged more slowly than in nitrogen-rich environments. Whole-genome sequencing of 34 resistant strains revealed that mutations conferring resistance differed between nitrogen-rich and nitrogen-starved conditions. Nitrogen starvation selected for mutations predominantly in glycosyltransferase genes, which are associated with cell surface modifications. In contrast to resistant strains isolated under nitrogen-replete conditions, which exhibited impaired heterocyst formation, resistant strains selected under nitrogen starvation maintained their ability to form functional heterocysts and persist in nitrogen-limited environments. Our findings suggest that nitrogen availability influences the evolutionary trajectory of phage resistance, favoring mechanisms compatible with nitrogen fixation under nitrogen starvation. These results provide new insights into the ecological resilience of nitrogen-fixing cyanobacteria under phage predation and demonstrate that nitrogen availability affects the cost of resistance, evolutionary trajectories, and resistance mechanisms.

## Introduction

Cyanobacteria are one of the central primary producers on Earth. Due to their ability to perform photosynthesis and the capacity for some species to fix nitrogen, these microbes significantly influence biogeochemical cycles, including carbon and nitrogen cycles [1]. However, cyanobacteria can also form dense and often toxic blooms in freshwater, a worldwide phenomenon that has been increasing in the past decades [2, 3] and is predicted to intensify in the near future, including due to climate change and anthropogenic eutrophication processes [4–7]. Many of these cyanobacterial blooms are formed by diazotrophic (nitrogen-fixing) cyanobacteria, with the ability to fix nitrogen providing an advantage over other phytoplankton members that are unable to fix nitrogen [7], particularly when nitrogen levels are low. The most successful diazotrophic bloom-forming species is *Cylindrospermopsis raciborskii* [8]. This species, belonging to the *Nostocales* order, is known for its invasiveness [8] and potential toxicity [9], forming blooms in both tropic and temperate regions of all continents except Antarctica [10].

Nitrogen fixation is a process in which atmospheric nitrogen (N_2_) is converted into ammonia by the nitrogenase enzyme complex. Since this complex is irreversibly inactivated by oxygen [1], nitrogen fixation cannot be performed simultaneously with the oxygen-producing photosynthetic process. Thus, nitrogen fixation in filamentous cyanobacteria of the *Nostocales* order is facilitated in specialized cells called heterocysts, whereas photosynthesis is performed in the vegetative cells. The heterocyst cell envelope is thicker and more complex than that of vegetative cells, restricting the diffusion of gases, including atmospheric O_2_, into the heterocyst cell [11, 12]. Although N_2_ fixation is an advantageous process under nitrogen starvation, it is energetically expensive [7, 13]. Therefore, nitrogen fixation and heterocyst cell differentiation are highly regulated by a complex web of genes, which are affected by the availability of fixed nitrogen [8].

Cyanobacterial blooms are controlled by various abiotic factors, such as nutrient availability and temperature [14]. Furthermore, biotic factors, such as grazing, microbial, and viral predation, are also likely important regulators of bloom dynamics, although their contributions are only starting to be elucidated [15, 16]. For example, cyanophages (viruses that infect cyanobacteria) are known to affect cyanobacterial community composition and genome evolution in multiple ways, including by killing cyanobacteria in a host-specific manner, which impacts population size and strain/species composition [17]. Furthermore, cyanophages can facilitate horizontal gene transfer, affecting cyanobacteria genome composition and function [18]. Finally, predation by cyanophages leads to the emergence of antiphage-resistant strains, which influences intra-species variation [19]. Therefore, cyanophages are a strong selective force and regulator of cyanobacteria population dynamics [20]. Although the role of cyanophages in regulating blooms remains unclear, previous studies showing that cyanophage abundance co-occurs with the decrease of bloom-forming cyanobacteria and with the increase of dissolved toxins released from lysed cyanobacteria [21–23] suggest that cyanophages may control bloom dynamics.

Although cyanophages can kill cyanobacteria, cyanobacteria are known to rapidly become resistant against phage infection [24–26]. Genomic analysis of freshwater cyanobacteria revealed that they possess many Clustered Regularly Interspaced Short Palindromic Repeats (CRISPR)-Cas systems [27, 28] and additional defense mechanisms [28] that protect against cyanophage predation. For example, multiple resistance mechanisms are mediated by genetically encoded reduction in the adsorption of the phage to the cell surface [24, 25, 29–31], whereas for some strains resistance seems to be modulated by intracellular mechanisms [29, 32–34]. However, resistance to phages is usually associated with adaptive costs (or tradeoffs) for the cyanobacteria, such as reduction in the growth rate [24, 35, 36], higher susceptibility to other viruses [24], a reduction in the relative fitness under particular environmental conditions [26, 37, 38], or altered phenotypes for resistant strains [17, 39].

Recently, we reported an additional cost of phage resistance in nitrogen-fixing cyanobacteria, in which strains resistant to cyanophages that were isolated in a nitrogen-rich environment had a decreased ability (and sometimes complete inability) to produce heterocyst cells when transferred into nitrogen-depleted media [26]. In addition, the heterocyst cells that were induced by the resistant strains were defective and unable to fix atmospheric nitrogen. These data show that there is a clear tradeoff between resistance and nitrogen fixation, as phage-resistant strains evolved under nitrogen-rich conditions have a substantially decreased ability to survive once nitrogen levels are low. However, nitrogen-fixing cyanobacteria proliferate in nature under nitrogen starvation and form blooms, and thus, the reduced ability to fix nitrogen under these conditions may prevent the survival of phage-resistant strains in nature. Therefore, it remains unclear whether and how nitrogen-fixing cyanobacteria can evolve resistance without compromising nitrogen fixation under bloom-inducing nitrogen starvation conditions. Here, we address this question by analyzing the evolution of phage resistance in nitrogen-fixing cyanobacteria (*Nostoc* PCC 7120 and *C. raciborskii*) under nitrogen starvation and comparing the distinct evolutionary pathways of phage resistance in cyanobacteria under nitrogen-limited and nitrogen-rich conditions. We show that multiple strains of heterocystous cyanobacteria resistant to phages can evolve under nitrogen starvation, albeit this process is slower than in nitrogen-rich medium. Furthermore, we characterize the mutations and phenotypes involved in phage resistance and demonstrate that nitrogen starvation selected for resistance mutations predominantly associated with cell surface modifications without impacting nitrogen fixation, which contrasts with the evolution of resistance under nitrogen-rich conditions. Our findings provide insights into the adaptive strategies of nitrogen-fixing cyanobacteria and how these microbes balance phage resistance and nitrogen fixation in response to environmental conditions.

## Materials and methods

### Culturing conditions

Cyanobacterial strains were cultured in BG11_0_ medium, which contains nearly no nitrogen source [29], under a daily cycle of 10 h of darkness and 14 h of light, at a temperature of 24°C, with a photon flux density of 8–11 μmol/m^2^ s photons flux. For plating, cultures in the exponential growth phase were serially diluted 10-fold, and each dilution was mixed with BG11_0_ medium and low-melting-point agarose at a final concentration of 0.28%. The mixture was then poured into 90 mm Petri dishes. Single colonies formed within 4–5 weeks and were subsequently transferred to liquid BG11_0_ medium for further cultivation [24]. When relevant, BG11 [29] was used to simulate nitrogen-replete conditions.

### Cyanobacterial strains

The diazotrophic, heterocystous cyanobacteria *C. raciborskii* strain KLL07 and *Nostoc* PCC 7120 were used as model organisms. *Cylindrospermopsis raciborskii* KLL07 was originally isolated from Lake Kinneret, Israel [40], where it has been responsible for the majority of summer blooms since the mid-1990s, particularly under nitrogen-limited conditions [41]. The genome of this strain has been fully sequenced [42]. *Nostoc* PCC 7120 is a well-characterized model cyanobacterium with a fully sequenced genome [43].

### Cyanophages

*Cylindrospermopsis raciborskii* was challenged with three phages: Cylindrospermopsis phage Cr-LKS4, Cr-LKS5, and Cr-LKS6. These phages were isolated from Lake Kinneret during a *C. raciborskii* bloom, and their genomes have been fully sequenced [26]. The three phages have high genomic similarity to the CrV phage [44].

*Nostoc* PCC 7120 was infected with two cyanophages: Anabaena phage A-4L and Cyanophage AN-15. A-4L was originally isolated in 1972 in Leningrad, USSR [45], and belongs to the *Saffermanviridae* family [46]. AN-15 was isolated in 1981 in south-central Michigan, and it has a long contractile tail, unlike the short tail of A-4L [47]. These phages were obtained from the ATCC culture collection. For infections, *Nostoc* 7120 cultures were exposed to A-4L, AN-15, or a combination of both phages.

### Isolation of strains resistant to phages under nitrogen starvation

Phage-resistant cyanobacterial strains were isolated using two selection methods, as described previously [26]: selection in semi-solid medium and selection in liquid medium.

For selection in semi-solid medium, homogeneous cyanobacterial cultures, each derived from a single colony, were serially diluted and plated in a semi-solid BG11_0_ medium (low-melting point agarose) with or without phage exposure (control). Colonies that emerged were transferred into liquid BG11_0_ medium in 96-well plates, with gradual increases in culture volume.

For selection in liquid medium, cyanobacterial cultures were infected with cyanophages in 96-well plates. Each well contained 170 μl of cyanobacterial culture inoculated with 30 μl of the specific phage lysate or 30 μl of BG11_0_ medium as a negative control. Growth was monitored using chlorophyll autofluorescence as a proxy for cell concentration, measured with a Synergy 2 plate reader (BioTek) at an excitation wavelength of 440 nm and an emission wavelength of 680 nm. Surviving cultures were serially diluted and plated in a semi-solid medium to obtain single colonies, which were then transferred to liquid medium for further cultivation.

To preserve the isolated strains, 3 ml of each culture were centrifuged, the supernatant was removed, and the pellet was stored at −80°C for future DNA extraction. Additionally, 10 ml of each strain were centrifuged at 3500 rpm for 5 min, the supernatant was replaced with 1 ml of 7.5% Dimethyl Sulfoxide (DMSO) in BG110 medium, and the samples were stored at −80°C for long-term preservation.

### Validation of phage resistance

To confirm phage resistance, each isolated strain was re-infected with the phage it was originally selected against. Three biological replicates were used per strain. For each replicate, 180 μl of cyanobacterial culture was inoculated with 20 μl of the corresponding cyanophage in a 96-well plate. As a negative control, three wells of the resistant cyanobacteria were mock-infected with 20 μl of BG11_0_ medium instead of cyanophage. The susceptible ancestral strain of each resistant isolate was also infected with the corresponding cyanophage in the same manner and served as a positive control.

### Filament length and heterocyst cell frequency assessment

Morphological comparisons between phage-resistant strains and their ancestors were conducted using an epifluorescence microscope (Nikon Eclipse Ti-E). *Nostoc* 7120 filament length was assessed by imaging at least 100 filaments from each strain (resistant and ancestral) across three biological replicates. Measurements were performed using the microscope’s software and normalized by dividing the total filament length by 4 μm, the average cell length of *Nostoc* 7120. Heterocyst frequency was determined by manually counting the number of heterocysts per filament. Filaments that contained 20 cells or less were probably the result of recent fragmentation of longer filaments and mostly contained a single heterocyst and a changing number of vegetative cells, which substantially affected the frequency of heterocysts per filament. Therefore, these filaments were not used for the heterocyst frequency analysis.

For *C. raciborskii*, we calculated the percentage of filaments containing one or two apical heterocysts, based on at least 30 filaments per biological replicate.

### Gene expression of *nifH*

To assess differences in *nifH* gene expression between resistant *Nostoc* 7120 substrains and their ancestral, susceptible wild-type (WT) paired controls, we followed the protocol described previously [26]. RNA was extracted using the Zymo Quick-RNA MiniPrep Plus Kit (ZR-R1057) with ZR BashingBead Lysis, followed by an additional DNase I treatment (TURBO DNA-free AM1907) to remove genomic DNA contamination. RNA concentration and purity were assessed using a Qubit 2.0 fluorometer (Invitrogen). Complementary DNA (cDNA) synthesis was performed using the High-Capacity cDNA Reverse Transcription Kit (AB-4374966).

Quantitative reverse transcription PCR was conducted using the LightCycler 480 SYBR Green I Master mix (Roche) on a QuantStudio Real-Time PCR system (Applied Biosystems). Fluorescence data were recorded using QuantStudio Design and Analysis Software (version 1.5.1). To confirm amplification specificity, melting curve analysis was performed.

Standard curves were generated using plasmids containing cloned target sequences, allowing quantification of gene copy numbers. PCR products of *nifH* and the reference gene *rnpB* were cloned into the pGEM-T plasmid (Promega), and cDNA concentrations were measured using a Qubit 2.0 fluorometer. DNA concentrations were converted into genome copy numbers per milliliter using the DNA Copy Number Calculator (Thermo Fisher Scientific Inc.). The expression level of *nifH* was normalized to *rnpB*.

### Assessment of nitrogenase activity

Nitrogen-fixation rates were measured as described previously [26, 41] by assessing nitrogenase enzyme activity using the acetylene reduction assay. Culture samples of 5 ml were placed into 28-ml serum bottles, sealed with a rubber septum, and reinforced with an aluminum closure. The bottles were flushed for 5 min with argon followed by the addition of C_2_H_2_ (20% of head space volume), and incubated for 48 h under controlled light and temperature conditions. After incubation, samples were analyzed immediately for C_2_H_4_ accumulated in the sample by injection of 1 ml of the headspace gas to a Gas Chromotography Flame Ionization Detector (Shimadzu) using Durapak phenyl isocyanate on 80/100 Porasil C in a 60 9 1/8″ column (Supelco), calibrated with ethylene (100 ppm standard, Supelco, Cat No: 22572; Sigma-Aldrich, Rehovot, Israel). Chlorophyll concentrations in all samples were determined by fluorometry after 90% acetone extraction using the method of Holm-Hansen *et al*. [48], and nitrogenase activity rates were normalized to the chlorophyll content in each sample, and averaged over two technical replicates for each biological replicate.

### Statistical analyses

To compare the recovery of *Nostoc* 7120 populations following phage infection, by the emergence of resistant populations over time, under different nitrogen conditions (with or without a fixed nitrogen source), recovery probabilities were plotted as Kaplan–Meier survival curves. Differences between curves were assessed using the Mantel–Cox log-rank test, implemented in GraphPad Prism (Version 10.4.0; https://www.graphpad.com/scientific-software/prism/).

To compare the time to recovery of *C. raciborskii* populations grown under nitrogen-rich vs. nitrogen-depleted conditions, a two-tailed Mann–Whitney U test was applied, as the data from nitrogen-rich conditions did not meet normality assumptions according to the Shapiro–Wilk test. This analysis was performed in R (version 4.4.2) [49].

Differences in filament length and heterocyst frequency between phage-resistant *Nostoc* 7120 substrains and their susceptible ancestors were also evaluated using two-tailed Mann–Whitney U tests (Supplementary Table S1), following rejection of normality by the Shapiro–Wilk test. Analyses were performed in R (version 4.1.2) [49].

To assess differences in the proportion of filaments with heterocysts vs. without heterocysts between resistant and susceptible *C. raciborskii* strains, two-tailed t-tests were used when data were normally distributed; otherwise, the nonparametric Mann–Whitney U test was applied. The latter was used for all substrains derived from colonies D and RNE3 (Supplementary Table S1). All analyses were conducted in R (version 4.4.2) [49].

To evaluate differences in normalized *nifH* expression and nitrogenase activity between resistant and susceptible *Nostoc* 7120 substrains, two-tailed t-tests or Mann–Whitney U tests were used depending on whether the data passed the Shapiro–Wilk normality test. The nonparametric test was used for *nifH* expression of RNA1 and RNA3 (Supplementary Table S1). Analyses were performed using R (version 4.4.2) [49]. For all the above comparisons between phenotypes in the resistant strains and their susceptible ancestors, we used the Bonferroni adjustment to account for multiple comparisons [50].

### Whole-genome sequencing

Genomic DNA from phage-resistant strains and their paired ancestral (susceptible) colonies was extracted using the Presto Mini gDNA Bacteria Kit (Geneaid). Multiplexed DNA libraries were prepared using the Nextera DNA Flex Library Prep Kit following the protocol described previously [51]. Whole-genome sequencing of both resistant and ancestral strains was performed on a HiSeq System (Illumina).

### Mutation calling

Mutation detection was conducted using the *breseq* pipeline [52]. The reference genome for *Nostoc* PCC 7120 (GenBank accession: NC_003272, NZ_AP003581-AP003599) was used for alignment. Reads from all resistant strains were mapped to the reference genome and compared with their respective ancestral strains. To identify mutations specific to the resistant strains, shared mutations present in both resistant and ancestral genomes were filtered out. All detected mutations were manually inspected and validated using the Integrative Genomics Viewer (version 2.9.0) [53].

### Gene annotation

Functional annotation of the mutant genes was performed using a combination of functional predictions from the Integrated Microbial Genomes (IMG) database [54] and Protein Basic Local Alignment Search Tool (BLASTP) searches. Clusters of Orthologous Groups (COGs) and protein family classifications were retrieved from the IMG database.

Genes associated with cell surface modifications were identified based on their functional annotations, the presence of transmembrane helices (TMHs) as predicted by IMG, the functions of neighboring genes, and their functional category and cellular component classification in ALCOdbCyano (http://alcodb.jp/cyano/). Genes containing more than two TMHs were classified as membrane-associated, with additional verification based on their functional annotation to determine whether they were specifically linked to the outer membrane. Regulatory genes were identified based on functional annotation and categorized accordingly.

### Core gene identification

Core genes were identified using the CoreGenes 5.0 tool [55]. Homologous genes were identified in *Nostoc* PCC 7120 and compared to genomes of 18 additional *Nostocales* strains available in the NCBI GenBank database (Supplementary Table S2).

### Glycosyltransferase gene quantification

Glycosyltransferase genes were identified by searching for genes annotated with the term “glycosyltransferase” in the COG database, using the functional search tool in IMG [54]. Comparative analyses included genomes randomly selected from cyanobacterial orders available in the IMG dataset, in addition to *Nostoc* PCC 7120.

## Results

### Dynamics of *Nostoc* 7120 phage infection under nitrogen starvation

**Figure 1:**
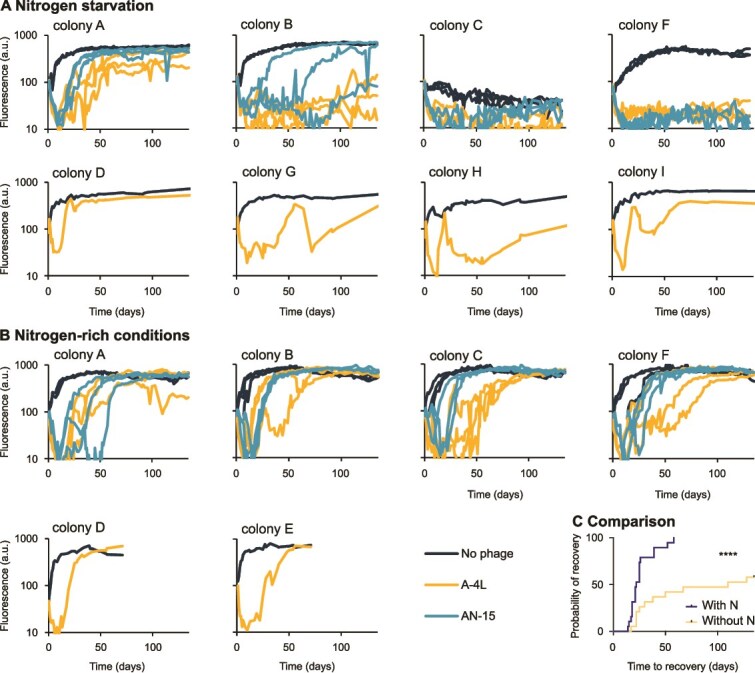
Population dynamics of *Nostoc* 7120 infected by phages A-4L or AN-15 in nitrogen-starvation (A) vs. nitrogen-replete conditions (B), showing relative cell density, estimated by chlorophyll a autofluorescence (a.u., arbitrary units) over time in individual populations and a Kaplan–Meier curve of recovery probability from 19 infected populations per condition; *P* value ^****^ < .00001.

To assess the evolution of phage resistance in nitrogen-fixing cyanobacteria under nitrogen starvation, we started by exploring the dynamics of infection of the model heterocystous cyanobacterium *Nostoc* PCC sp. strain 7120 (*Nostoc* 7120) by cyanophages A-4L or AN-15. When a culture of cyanobacteria is infected by a lytic phage, most cells are usually lysed and the population collapses. However, cells that gained resistance to the phage survive and eventually enable the recovery of the infected population. We followed these dynamics starting with eight populations of *Nostoc* 7120, each originating from a separate single colony, which were grown in nitrogen-depleted medium and then infected by phage lysates (A-4L or AN-15). Although all populations collapsed 3–4 days postinfection (Fig. 1), their recovery time varied greatly both between and within populations evolved from different colonies (Fig. 1). For example, cultures evolved from colony A, infected by A-4L, recovered 22–50 days postinfection, whereas cultures evolved from colony B infected with the same phage recovered more slowly (110–128 days postinfection) or did not recover at all (during the 135 days analyzed) (Fig. 1A). None of the replicates that evolved from colony F (infected with either A-4L or AN-15) was able to recover during the 135 days of the experiment. The uninfected control originating from colony C collapsed under nitrogen depletion even in the absence of phage infection (and thus is discussed separately; see Supplementary Text). Moreover, some of the infected cultures collapsed a second time after the initial recovery (see, e.g. colony G in Fig. 1A), suggesting the existence of a co-evolutionary arms race between the phage and host (see Supplementary Text). A comparison of recovery rates revealed that populations were more likely to recover in nitrogen-rich medium than under nitrogen starvation (100% vs. 68%). Additionally, among populations that successfully recovered, the average recovery time was longer under nitrogen starvation (42.3 days) compared with nitrogen-rich conditions (29.4 days; Fig. 1). However, there was substantial variability in recovery times (ranging from 14 to 58 days in nitrogen-rich medium and from 17 to 131 days in nitrogen-depleted medium). Collectively, these findings demonstrate that phage-resistant strains can evolve under nitrogen-starvation conditions, albeit at lower frequency and more slowly than in nitrogen-rich media (Fig. 1C; $P<.0001$).

### Physiology of the resistant strains

Previously, we found that phage-resistant strains evolved under nitrogen-rich conditions have a reduced ability to fix nitrogen and/or to survive nitrogen starvation. To understand if phage resistance evolved under nitrogen starvation is also associated with tradeoffs, we isolated and characterized the physiology of 34 *Nostoc* strains resistant to A-4 L and/or AN-15 (Supplementary Table S3). Most of these strains (24/34) were isolated during the initial recovery of the infected population (Supplementary Table S3), whereas the remaining (10/34) strains were isolated after a secondary recovery. These strains are referred to as the first- and second-generation resistant strains, respectively. Susceptibility tests showed that the vast majority (28/34) of the strains were fully resistant to the selecting phage (infection dynamics similar to the uninfected treatment, e.g. Supplementary Fig. S1B), whereas some (6/34) were partially resistant (the collapse of the infected population was delayed and partial, in comparison to that of the susceptible strain, e.g. Supplementary Fig. S1C  vs.  A and Supplementary Table S3).

**Figure 2:**
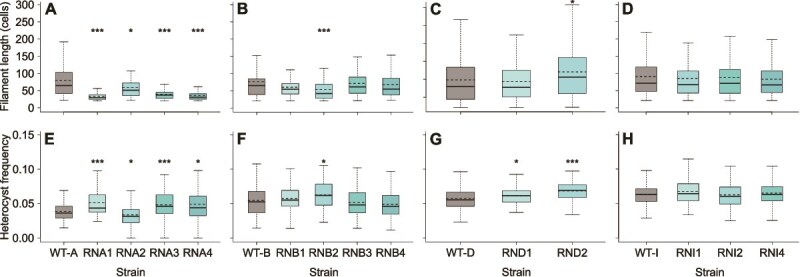
Distribution of filament lengths (A–D) and heterocyst frequency per filament (E–H) of resistant strains evolved from colonies A (A and E), B (B and F), D (C and G), and I (D and H) in comparison to the susceptible ancestral strain (WT); adjusted *P* value: ^*^ < .05 and ^***^ < .001; dashed line marks the average of the values identified for each strain; see Supplementary Table S1 for exact *P* values, biological replicate numbers, and mean values.

In addition to phage susceptibility, we also characterized a subset of the resistant strains (13/34) with regard to filament length, which may be related to nitrogen-fixation ability [56, 57]. Eight of the analyzed resistant strains (8/13) had a distribution of the length of their filaments similar to that of the WT parental strain, whereas the other strains (5/13) had filaments shorter by 26%–58% (Fig. 2A–D). Most of the analyzed resistant strains had heterocyst frequency similar (6/13) or higher by 8%–32% (6/13) than that of their ancestors, whereas only one (1/13) had significantly lower frequency (by 13%; Fig. 2E–H).

**Figure 3:**
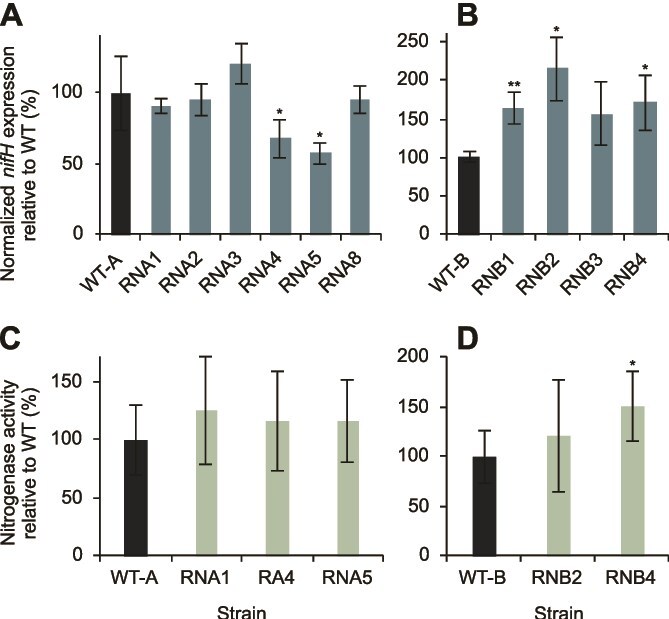
Expression of *nifH* gene (A and B) and nitrogenase activity (C and D), relative to the susceptible ancestor (WT), of the resistant substrains evolved of colony A (A and C) or B (B and D); the transcript levels of *nifH* values are normalized to the transcript levels of *rnpB*; data shown are average ±SD of three to seven biological replicates; ^*^$P<.05$, ^**^$P<.01$, ^***^$P<.001$; see Supplementary Table S1 for exact *P* values, biological replicate numbers, and mean values.

We showed previously that the tradeoff between resistance to phages and heterocyst functionality in strains evolved under nitrogen-rich conditions can be manifested by reduced activity of the enzyme that performs nitrogen fixation, nitrogenase, as well as by reduced expression of the *nifH* gene, which encodes a critical component of the nitrogenase complex [26]. Therefore, we also assessed *nifH* expression and nitrogenase activity in a subset of the strains selected for resistance under nitrogen starvation. In agreement with the results of the heterocyst frequency analysis, we observed that the majority of analyzed resistant strains (8/10) had no reduction in *nifH* expression, whereas four (4/10) of these strains even showed a significant increase in *nifH* expression (Fig. 3A and B). Moreover, none of the analyzed resistant strains showed a reduction in nitrogenase activity (Fig. 3C and D). Although this activity varied across the analyzed resistant strains, all (5/5) showed substantial nitrogenase activity (Fig. 3C and D). Collectively, these data show that most phage-resistant strains that evolved under nitrogen starvation conditions retain their ability to produce heterocysts, express *nifH*, and exhibit nitrogenase activity, demonstrating that they have maintained their ability to fix nitrogen.

### Mutations associated with phage resistance

Despite the tradeoff between resistance to phages and nitrogen fixation that we recently observed when phage selection was applied in nitrogen-rich conditions [26], nitrogen starvation conditions did not prevent the appearance of phage resistance. To better understand the differences between strains selected for phage resistance under nitrogen-rich or nitrogen-depleted conditions, we sequenced the genomes of the 34 *Nostoc* 7120 strains resistant to phage A-4L and/or AN-15 evolved under nitrogen starvation, as well as the genomes of their susceptible ancestors (Supplementary Table S3), and compared these to the genomes of the strains previously evolved under nitrogen-rich conditions [26].

The resistant strains that evolved under nitrogen starvation evolved in parallel from six susceptible clones, three of which were in common with those previously reported (WT-A, WT-B, and WT-D) and sequenced previously [26]. Our analysis of the 34 resistant strains that evolved under nitrogen starvation revealed 203 mutations, of which 137 were common with their ancestors (Supplementary Table S4) and 66 were specific to the resistant strains. For seven of the 34 resistant strains that were sequenced, coverage was low and incomplete, so these genomes may encode additional mutations associated with resistance. In addition, four strains that evolved under nitrogen starvation conditions with no phage selection were used as a control for nutrient-specific mutations (see Supplementary Text).

Each resistant strain of *Nostoc* evolved under nitrogen starvation carried 1–13 of the 66 resistant-specific mutations. Of these 66 mutations, 39 were SNPs, 16 were insertions, and 11 were deletions. Eight of these mutations were intergenic, and of the mutations found within coding regions, 11 were silent, 20 were missense, and 27 were nonsense (Supplementary Table S3). These mutations affected 54 genes, the majority (91%) of which were non-core genes (Supplementary Table S5), suggesting that, similarly to previous studies [26, 58], phage selection promotes host genetic variability. Nearly half of the affected genes with predicted function were either cell surface-related (26%) or regulatory genes (21%).

We compared the mutations found in the first- and second-generation resistant strains (Fig. 1A). Strains isolated from the secondary recovery carried significantly ($P=.001$) more mutations per strain than those isolated from the first recovery (average of 6.8 vs. 2.7, respectively). Although the number of mutations per strain varied greatly (2–13 vs. 1–8; Supplementary Table S3), strains carrying a single resistance-related mutation were isolated only from the first recovery. In total, 46 and 21 resistance-specific mutations were identified in the first- and second-generation resistant strains, respectively. Only one of the mutations present in the second-generation strains was common with the mutations a found in the first-generation strains: a deletion leading to a frameshift in *alr4493* that encodes for glycosyltransferase. This specific mutation appeared independently in four of the analyzed lineages (A, B, D, and I) in multiple first-generation strains, as well as in four second-generation strains descended from lineage I (Supplementary Table S3), and thus it is unclear whether the mutations in the first and second generations are independent.

### Genes in which mutations evolved multiple times

**Figure 4:**
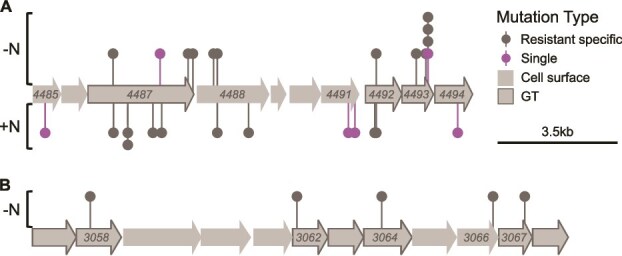
Genomic neighborhood of two cell surface-related gene clusters enriched with mutations specific to the resistant strains: *alr4485*–*alr4494* (A) and *alr3057*–*alr3068* (B); gray arrows represent cell surface-related genes, and those outlined and marked with GT encode specifically for glycosyltransferases; mutations shown above the genes were identified in strains isolated in nitrogen starvation conditions (this study), whereas those shown below the genes were previously identified in nitrogen-replete conditions [26].

Of the 54 genes affected by resistance-related mutations, four genes had mutations that evolved multiple times (Fig. 4). All four genes encode for different glycosyltransferases, which take part in the modification of the *Nostoc* cell surface. Moreover, all of these mutations were non-synonymous, and most of them (13/16) were nonsense mutations, suggesting that the function of these genes was abolished. The first gene (*alr4346*) had four mutations that evolved in parallel in five strains. The other three genes (*alr4487*, *alr4488*, and *alr4493*) had four, two, and six mutations that evolved in parallel, in 10, 6, and 13 strains, respectively. These genes (as well as *alr4492,* which had a single mutation in this study) belong to a gene cluster enriched with cell surface-related genes (Fig. 4A). Multiple resistance-related mutations were identified in this cluster in a previous study, in which resistant strains were isolated in nitrogen-rich medium [26]. However, most of these mutations (11/13; including *alr4492*) differed in the strains isolated in nitrogen-rich [26] or nitrogen-depleted (this study) conditions (Fig. 4A). The strains carrying the other two mutations had various compositions of additional mutations in other genes, which may affect the phenotypes of these strains. Although we identified six mutations in *alr4493*, no mutation was identified in this gene by a previous study [26].

In addition to the multiple mutations found in the *alr4485*–*alr4494* genomic region, we identified five additional mutations in the *alr3057*–*alr3068* genomic region. These five mutations (in *alr3058*, *alr3062*, *alr3064*, *alr3066*, and *alr3067*) are also in a gene cluster enriched with cell surface-related genes (Fig. 4B). All 12 genes in this gene cluster are cell surface-related, with seven of them encoding glycosyltransferases. Four of the mutated genes encode for glycosyltransferases (Fig. 4B), whereas the fifth gene encodes for Polysaccharide pyruvyl transferase (Supplementary Table S3).

Six of the genes affected by resistance-related mutations (*all2170*, *alr3683*, *alr4346*, *alr4487*, *alr4488*, and *alr4492*) had mutations in both strains isolated under nitrogen starvation and in nitrogen-rich medium [26]. Only one of these mutations (in *alr3683*), found in strains with the same ancestor (RNA1 and RA2), was identical in the strains isolated in the different conditions, suggesting they appeared before the ancestral population was transferred into the different conditions. Other mutations were found in strains with a different ancestor, or were in a different locus within the same gene, suggesting they evolved in parallel. Most of these mutations were found in genes or gene clusters enriched with resistance mutations as described above.

### Mutations conferring resistance

To better understand the links between mutations and phage resistance, we focused on resistant strains with a single mutation, as in these strains there is a higher chance that the mutations identified are directly involved in conferring resistance. Four resistant strains had a single mutation in their genome (Supplementary Table S3). One of these mutations was in a gene with unknown function (*asr4302*) and the others were in three genes encoding for different glycosyltransferases (*alr4487*, *alr4493*, and *all4933*). All four genes are noncore genes (Supplementary Tables S3 and S5). Moreover, the majority of the other resistant strains (with more than one mutation) had mutations in one (33%) or two (50%) genes encoding for various glycosyltransferases (Supplementary Table S3). Although this does not necessarily show that these mutations in the glycosyltransferase genes are those that confer the resistance in the strains carrying multiple mutations (except in three strains carrying mutations only in glycosyltransferase genes), these results further stress the importance of mutations in these genes in resistance selected under nitrogen starvation.

Of the four genes with a single mutation in a genome, mutations in *asr4302* and *all4933* were not found in any of the other resistant strains (or in strains isolated in replete nitrogen conditions [26]). The other two mutant genes (*alr4487* and *alr4493*) are located in the gene cluster (*alr4485*–*alr4494*) enriched with mutations specific to resistant strains isolated under both nitrogen-rich [26] and nitrogen-depleted (this study) conditions. However, mutations in *alr4493* were found only in this study, and although we identified multiple mutations in *alr4487* in this study and in [26], most of the other strains carried different mutations in *alr4487* and they were always accompanied by other mutations, suggesting that different mutations in the same gene and/or different mutant genes composition may alter the phenotype of the affected strain. Moreover, although the mutation in *alr4493* (insertion of A leading to a frameshift), which was close to the C terminus of the gene, conferred only partial resistance, some combinations of this mutation with mutations in other genes conferred full resistance, further stressing the importance of the exact mutation composition in determining the strain’s phenotype.

### Phage resistance in the bloom-forming species *C. raciborskii*

**Figure 5:**
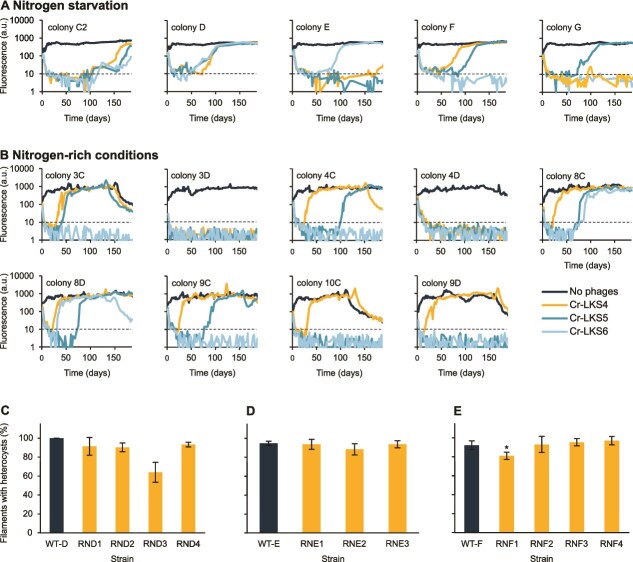
Populations of *C. raciborskii* infected by phages, or with no phage for control without (A) or with (B) fixed nitrogen source (NO_3_^–^); relative cell density was estimated by chlorophyll a autofluorescence (a.u., arbitrary units); percent filaments with heterocysts in resistant vs. susceptible ancestral strains of strains evolved from WT-D (C), WT-E (D), and WT-F (E); WT denotes Wild-type ancestral strain; see Supplementary Table S1 for exact *P* values, biological replicate numbers, and mean values.

We previously saw that the tradeoff between resistance to phages and heterocyst induction and functionality that emerges when phage selection is conducted under nitrogen-rich conditions is not restricted to *Nostoc* 7120 but also seen in the invasive cyanobacterium *C. raciborskii*, which is one of the most important freshwater invasive *Nostocales* species [8, 26]. Therefore, we assessed whether the appearance of phage resistance under nitrogen starvation is restricted to *Nostoc* 7120 or also shared by *C. raciborskii*. We thus infected 15 *C. raciborskii* cultures that originated from five colonies (evolved in parallel) with three phages (Cr-LKS4, Cr-LKS5, and Cr-LKS6), which are all double-stranded DNA phages with long non-contractile tails (previously classified as siphoviruses) [26]. We then compared the population dynamics of the infected cultures (Fig. 5A) with those of cultures originated from nine colonies grown in nitrogen-rich medium (Fig. 5B). Similarly to the results observed with *Nostoc* 7120, most of the *C. raciborskii* cultures (10/15) infected under nitrogen starvation recovered during the experiment (Fig. 5A). Although recovering time varied within the two treatments, recovery time was longer in the recovered cultures under nitrogen starvation compared with the nitrogen-rich conditions (128.18 vs. 54.64 days; $P=.003$). Moreover, although there was no difference in recovery from infection by the different phages under nitrogen starvation, we found that under nitrogen-rich conditions the speed and frequency of recovery was higher following Cr-LKS4 infection compared with the other phages (recovered in 7/9 infections vs. 5 or 2 in Cr-LKS5 and Cr-LKS6, respectively) (Fig. 5B).

In addition to infection dynamics, we also characterized a subset of the recovered strains with regards to phage resistance and morphology, which is indicative of nitrogen fixation ability. We isolated 11 strains from the recovered populations, and their resistance to the selecting phage was verified (Supplementary Table S6). All the isolated strains had a similar morphology to that of the susceptible ancestor, and most of their filaments carried the typical apical heterocysts (Fig. 5C–E). Most of the resistant strains (10/11) had a similar distribution of filaments carrying heterocysts to that of their susceptible ancestors (Fig. 5C–E). Moreover, the resistant strain that showed reduced heterocyst frequency had only a mild reduction (of 12%) in the percentage of filaments with heterocysts (Fig. 5E). Working with *C. raciborskii* and its phages was technically challenging, and thus as in our previous study [26], additional physiological characterization of the resistant strains was not carried out. Moreover, since in Kolan *et al*. [26] we were not able to identify resistant-specific mutations in the *C. raciborskii* strains isolated in nitrogen-rich conditions, here we did not sequence the genomes of the resistant strains isolated under nitrogen starvation. Nonetheless, together with the ability of these strains to proliferate under nitrogen starvation for >2 years, these results further suggest that similarly to the *Nostoc*-resistant strains, *C. raciborskii* is able to evolve and become resistant to phages under nitrogen starvation without losing its ability to fix nitrogen.

## Discussion

Cyanophages have a great impact on cyanobacterial community dynamics, both ecologically and evolutionary [18, 20]. For example, lytic cyanophages select for resistance by killing the susceptible individuals in the community and allowing the resistant individuals to thrive and takeover the population. In this process, cyanophages can select for different types of phage-resistant phenotypes [17]. Previously, we showed that heterocystous nitrogen-fixing cyanobacteria grown in a nitrogen-rich environment can recover from cyanophage infection by evolving resistance to the phage [26]. This resistance comes at a cost, manifested by a reduced ability to produce heterocyst cells and to fix nitrogen. As a result, such resistant strains have a limited ability to persist under nitrogen starvation. These nitrogen-fixing cyanobacteria tend to form blooms under low nitrogen conditions [41], so a reduced ability to fix nitrogen in such environments may affect their survival and their ability to bloom in nature. Therefore, here we studied the potential of such cyanobacteria to become resistant to phages under nitrogen starvation conditions, similar to those conditions that can be found during blooms.

We found that populations of both *C. raciborskii* and *Nostoc* 7120 are able to recover from phage infection under nitrogen starvation by the growth of strains resistant to the infecting phage. Cultures of both genera infected by various phages showed high variability in recovery capacity (both with regards to recovery frequency and speed), which is in agreement with the high variability in the number of mutations identified in the resistant *Nostoc* 7120 strains (1–8 mutations per genome in the first-generation mutants; Supplementary Table S3). Although we found differences in the infection and recovery dynamics of *Nostoc* 7120 and *C. raciborskii* under nitrogen starvation, both strains (belonging to different genera) showed a clear ability to become resistant to phages under nitrogen starvation, suggesting that this ability may be widespread among heterocystous cyanobacteria.

Our analyses also show that most of the *Nostoc-* and *C. raciborskii*-resistant strains isolated during nitrogen starvation retain their ability to produce heterocysts, and in most cases with a frequency similar to or even higher than that of the ancestral strain. Furthermore, although the resistant strains isolated in nitrogen-rich medium had a significant reduction in the expression of the nitrogenase-encoding *nifH* gene and in nitrogenase activity [26], the majority of the analyzed *Nostoc*-resistant strains evolved under nitrogen starvation had no significant reduction in *nifH* expression nor nitrogenase activity, demonstrating that these strains have retained their ability for nitrogen fixation. Moreover, all *Nostoc* 7120- and *C. raciborskii*-resistant strains were grown for 2–3 years in nitrogen starvation conditions, implying that the heterocysts are functional. These results suggest that at least a subset of the resistance-conferring mutations that evolved during nitrogen starvation in heterocystous cyanobacteria do not have a pleiotropic effect on the ability to fix nitrogen. Therefore, these data suggest that strains of nitrogen-fixing cyanobacteria that are resistant to phage infection can potentially evolve during cyanobacterial blooms. These findings raise the question of why, in our previous study, we did not identify any resistant strains with no tradeoff between phage resistance and nitrogen fixation in strains isolated in nitrogen-rich medium. Although additional studies are needed to address these differences, they could result from distinct cell surface composition of the resistant strains in the different environments, or a potential growth disadvantage in nitrogen-rich medium for the strains isolated under nitrogen starvation.

Our comparison of the mutations found in resistant strains selected in nitrogen-rich vs. nitrogen-depleted conditions revealed a clear difference in mutation profiles. There is almost no overlap in mutations between the two conditions, even though many resistant strains in both conditions evolved from the same ancestral lines and despite the mutation overlap observed within each condition. In both environments, we identified mutations in a gene cluster enriched with cell surface-related genes, with some mutations even affecting the same gene across conditions. However, key differences emerged, particularly with mutations in nitrogen-rich and nitrogen-depleted conditions generally affecting different sites or occurring in distinct genetic contexts (i.e. alongside mutations in different genes). Additionally, the gene *alr4493* harbored multiple mutations exclusively in nitrogen-depleted conditions, whereas other genes, such as *alr4485*, *alr4491*, and *alr4494*, exhibited multiple mutations only in nitrogen-rich conditions. These findings suggest that nitrogen availability influences the fitness landscape of phage resistance evolution, favoring mechanisms that are compatible with nitrogen fixation under starvation and leading to distinct genomic and phenotypic outcomes depending on nutrient conditions. Furthermore, in nitrogen-depleted conditions, there was a pronounced enrichment of mutations in glycosyltransferase genes. Although both conditions showed a general enrichment of mutations in cell surface-related genes, glycosyltransferase mutations were especially prominent under nitrogen starvation. These findings are aligned with previous studies reporting that mutations in various cell surface-related genes are associated with resistance to phages in both heterocystous [26] and unicellular cyanobacteria [24, 59], but suggest that selecting for resistance in heterocystous cyanobacteria under nitrogen starvation may particularly favor mutations in glycosyltransferase genes. Although the reasons for the specific involvement of glycosyltransferase need to be investigated in more detail, this may be because mutations in other cell–surface related genes can also lead to increased permeability of the heterocyst cell wall, which would lead to nitrogenase inactivation by oxygen penetration and reduced fitness of the resistant strains under nitrogen starvation. Although a few specific glycosyltransferase genes are essential for the differentiation and functionality of heterocyst cells [60, 61], *Nostoc* PCC 7120 (similarly to other filamentous bacteria) carries dozens of glycosyltransferase genes (Supplementary Table S7), suggesting that mutations in some of these may provide protection against phages without interfering with the permeability of the heterocyst cells.

The nature of the observed mutations also correlated with broader functional effects on the cells. For example, resistant strains selected in nitrogen-depleted medium displayed no pronounced phenotypes, which contrasted with the complete loss of heterocyst induction or other microscopic abnormalities observed in resistant strains selected in nitrogen-rich conditions. These observations suggest that nitrogen availability shapes specific adaptive strategies, possibly due to selective pressure favoring nitrogen-fixation compatibility in resistant strains under nitrogen starvation.

To conclude, although there is a known tradeoff between nitrogen fixation and resistance to phages in heterocystous cyanobacteria [26], nitrogen-fixing cyanobacteria resistant to phages can evolve and thrive under nitrogen starvation. These strains are able to produce heterocysts and fix atmospheric nitrogen, which can be advantageous in nature under nitrogen starvation conditions, such as during bloom formation. Nitrogen starvation seems to select for specific mutations, mostly in glycosyltransferase genes that probably modify the receptors of the phage, without impairing the function of heterocysts. These findings suggest that the tradeoff between nitrogen starvation and resistance to phages drives genome evolution of heterocystous cyanobacteria, even though it does not prevent the appearance of strains resistant to phages under conditions (nitrogen starvation) promoting blooms of these strains in nature. Moreover, these findings provide evidence for the crucial effect environmental conditions have on the evolutionary trajectory of phage resistance in cyanobacteria.

## Supplementary Material

MH_Supplementary_material_Res_AC_wraf180

Table_S3_wraf180

Table_S4_wraf180

Table_S5_wraf180

## Data Availability

The sequencing raw data (FASTq files) of all strains described in this manuscript have been deposited in GenBank under the accession numbers SAMN50549028-SAMN50549073.
