# Supplementary material for: Nitrogen availability shapes evolution of phage resistance in cyanobacteria
Source: ISME J. 2025 Aug 18;19(1):wraf180. doi: 10.1093/ismejo/wraf180 (PMC12507012; doi:10.1093/ismejo/wraf180)
Supplement: MH_Supplementary_material_Res_AC_wraf180 [file mh_supplementary_material_res_ac_wraf180.docx]

**Supplementary text:**

**Resistant strains evolved in rich nitrogen medium from a heterocyst-less ancestor**

During the infection dynamics experiments, we used uninfected cultures of the susceptible ancestors as a control for the host growth (Fig. 1). While all the uninfected cultures grew in nitrogen rich medium, one of the ancestral strains (WT-C) did not grow in the nitrogen poor medium, and none of its infected sub-cultures recovered. This suggested that this strain may have a reduced ability to fix nitrogen. To assess the source for this phenotype, we visualized the filaments of WT-C after 48 hours in nitrogen depleted medium. The other ancestral strains induced functional heterocyst cells under the same conditions [1], however, the visualization of WT-C (Fig. S2A) showed that this strain does not induce any heterocysts. Moreover, the expression of *nifH* in this strain, in the above nitrogen starvation conditions, was significantly lower than that of an additional WT strain (close to no expression; Fig. S2B). We then sequenced the genome of WT-C and compared it to the published genome of *Nostoc* 7120. WT-C had 99 mutations common to the other WTs (A, B, D, E, F, G, H, and I) and six additional unique mutations. Two mutations were in intergenic regions, and the four remaining mutations were in coding regions and included three SNPs and a single deletion (Table S3). One of the SNPs led to a stop codon in the gene alr2338 that encodes for SepJ (septal junction formation protein) which is located upstream to *hetR*, an essential regulator of the heterocysts differentiation process [2]. alr2338 was shown to have an important part in diazotrophic growth and in heterocysts maturation in *Nostoc* 7120 [3, 4]. Nayar et al. showed that inactivation of alr2338 resulted in filaments that induce immature heterocysts which express the Fix^-^ phenotype (lack of ability to fix N_2_ even in anaerobic conditions). Since WT-C had additional five unique mutations, we suggest that the mutation in *sepJ* is involved in the inability of this strain to fix N_2_, yet, we cannot exclude the option that other mutations are involved as well.

**Phage-host coevolution**

The initial recovery of some of the *Nostoc* populations was followed by an additional cycle of collapse and recovery. This may indicate that shortly after the first cycle of phage infection followed by the selection for resistance, phage mutants with the ability to infect the 1^st^ generation of resistant strains have evolved, resulting in population collapse. The second recovery of these populations was due to the selection for resistance to the evolved phage (the 2^nd^ generation of resistant strains). While 2^nd^ generation mutants carried significantly more mutations than the 1^st^ generation strains, they had nearly no overlap in mutations identity, and thus were probably evolved from other resistant strains that were in low frequency during the isolation of the 1^st^ generation strains. A similar pattern of arms race was seen in the cyanophage LPP-1 and its host *Plectonema boryanum* [5]. However, in other cyanophages, such as LPP-2 infecting *P. boryanum* [5] or different phages infecting the marine cyanobacteria *Prochlorococcus* sp. MED4 [6], the phage was unable to evolve host-range mutants that are able to infect the resistant strains.

**Nutrient-specific mutations**

In this study the resistant strains were selected using two selective pressures simultaneously: phage infection and nitrogen starvation. In order to assess whether the mutations in the resistant strains confer selective advantage under nitrogen starvation conditions and regardless of phage selection, we isolated four substrains that evolved under nitrogen starvation with no phage selection (in a similar timeframe as the resistant strains). These substrains evolved from the three ancestral colonies A (AN), B (BN) and F (F1N and F2N). The evolved substrains carried 0-4 mutations per genome (Table S7; AN had no mutations). Two of the mutant strains had mutations in the same locus (F2N, in *all3278*) or in other loci within the same gene (F1N, in *all1304*) as in the ancestral susceptible strains (Table S3). None of the genes carrying mutations in these substrains had mutations specific to the resistant substrains (Table S4). These results suggest that none of the mutations in the resistant strains could be classified as nutrient-specific mutation, however, this may result of the low number of susceptible strains sequenced.

**Supplementary Tables:**

**Table S1:** Statistical analyses in this study.

| Figure | Panel | Strain | Biological replicates | Average | Statistical test | *P* value | ^1^Asterisks | ^2^After Bonferroni adjustment |
| --- | --- | --- | --- | --- | --- | --- | --- | --- |
| 2 | A | WT-A | 107 | 79.8 |  |  |  |  |
|  |  | RNA1 | 61 | 33.4 | U-test | 2.37E-14 | *** | *** |
|  |  | RNA2 | 104 | 58.9 | U-test | 0.004194 | ** | * |
|  |  | RNA3 | 102 | 39.1 | U-test | 3.02E-13 | *** | *** |
|  |  | RNA4 | 91 | 35.8 | U-test | 6.72E-16 | *** | *** |
|  | B | WT-B | 111 | 76.2 |  |  |  |  |
|  |  | RNB1 | 108 | 60.2 | U-test | 0.04693 | * |  |
|  |  | RNB2 | 101 | 53.7 | U-test | 2.85E-05 | *** | *** |
|  |  | RNB3 | 106 | 71.2 | U-test | 0.7382 |  |  |
|  |  | RNB4 | 98 | 68 | U-test | 0.2997 |  |  |
|  | C | WT-D | 113 | 93.8 |  |  |  |  |
|  |  | RND1 | 125 | 121 | U-test | 0.895 |  |  |
|  |  | RND2 | 120 | 98.5 | U-test | 0.0205 | * | * |
|  | D | WT-I | 111 | 91 |  |  |  |  |
|  |  | RNI1 | 106 | 85.7 | U-test | 0.5817 |  |  |
|  |  | RNI2 | 112 | 88 | U-test | 0.7079 |  |  |
|  |  | RNI4 | 141 | 83 | U-test | 0.4636 |  |  |
|  | E | WT-A | 107 | 0.0385 |  |  |  |  |
|  |  | RNA1 | 61 | 0.0511 | U-test | 2.67E-05 | *** | *** |
|  |  | RNA2 | 104 | 0.0336 | U-test | 0.006732 | ** | * |
|  |  | RNA3 | 102 | 0.0482 | U-test | 2.98E-05 | *** | *** |
|  |  | RNA4 | 91 | 0.049 | U-test | 0.003741 | ** | * |
|  | F | WT-B | 111 | 0.0544 |  |  |  |  |
|  |  | RNB1 | 108 | 0.0571 | U-test | 0.1335 |  |  |
|  |  | RNB2 | 101 | 0.0625 | U-test | 0.00303 | ** | * |
|  |  | RNB3 | 106 | 0.0513 | U-test | 0.3462 |  |  |
|  |  | RNB4 | 98 | 0.0488 | U-test | 0.03838 | * |  |
|  | G | WT-D | 113 | 0.0573 |  |  |  |  |
|  |  | RND1 | 125 | 0.0617 | U-test | 0.009505 | ** | * |
|  |  | RND2 | 120 | 0.0684 | U-test | 4.2E-08 | *** | *** |
|  | H | WT-I | 111 | 0.0632 |  |  |  |  |
|  |  | RNI1 | 106 | 0.0675 | U-test | 0.1666 |  |  |
|  |  | RNI2 | 112 | 0.0625 | U-test | 0.4105 |  |  |
|  |  | RNI4 | 141 | 0.0651 | U-test | 0.5834 |  |  |

**Table S1 continued:**

| Figure | Panel | Strain | Biological replicates | Average | Statistical test | *P* value | ^1^Asterisks | ^2^After Bonferroni adjustment |
| --- | --- | --- | --- | --- | --- | --- | --- | --- |
| 3 | A | WT-A | 3 | 100% |  |  |  |  |
|  |  | RNA1 | 3 | 91% | U-test | 0.7 |  |  |
|  |  | RNA2 | 3 | 95% | t-test | 0.7883 |  |  |
|  |  | RNA3 | 3 | 121% | U-test | 0.4 |  |  |
|  |  | WT-A | 6 | 100% |  |  |  |  |
|  |  | RNA4 | 6 | 68% | t-test | 0.0021 | ** | * |
|  |  | RNA5 | 6 | 57% | t-test | 0.0021 | ** | * |
|  |  | RNA8 | 6 | 95% | t-test | 0.6991 |  |  |
|  | B | WT-B | 6 | 100% |  |  |  |  |
|  |  | RNB1 | 5 | 164% | t-test | 0.00142 | ** | ** |
|  |  | RNB2 | 5 | 215% | t-test | 0.00317 | ** | * |
|  |  | RNB3 | 5 | 157% | t-test | 0.03483 | * |  |
|  |  | RNB4 | 5 | 172% | t-test | 0.0099 | ** | * |
|  | C | WT-A | 7 | 100% |  |  |  |  |
|  |  | RNA1 | 7 | 124% | t-test | 0.2754 |  |  |
|  |  | RNA4 | 7 | 115% | t-test | 0.4618 |  |  |
|  |  | RNA5 | 7 | 116% | t-test | 0.3872 |  |  |
|  | D | WT-B | 6 | 100% |  |  |  |  |
|  |  | RNB2 | 7 | 121% | t-test | 0.2042 |  |  |
|  |  | RNB4 | 7 | 150% | t-test | 0.01315 | * | * |
| 5 | C | WT-D | 4 | 99.9% |  |  |  |  |
|  |  | RND1 | 4 | 91% | U-test | 0.06892 |  |  |
|  |  | RND2 | 4 | 90% | U-test | 0.02107 | * |  |
|  |  | RND3 | 4 | 64% | U-test | 0.02107 | * |  |
|  |  | RND4 | 4 | 93% | U-test | 0.02107 | * |  |
|  | D | WT-E | 4 | 94% |  |  |  |  |
|  |  | RNE1 | 4 | 93% | t-test | 0.7319 |  |  |
|  |  | RNE2 | 3 | 88% | t-test | 0.1979 |  |  |
|  |  | RNE3 | 4 | 93% | U-test | 0.6631 |  |  |
|  | E | WT-F | 4 | 92% |  |  |  |  |
|  |  | RNF1 | 4 | 81% | t-test | 0.009818 | ** | * |
|  |  | RNF2 | 4 | 93% | t-test | 0.8777 |  |  |
|  |  | RNF3 | 4 | 95% | t-test | 0.3308 |  |  |
|  |  | RNF4 | 4 | 97% | t-test | 0.1815 |  |  |

^1^*P* value: *<0.05, **<0.01, ***<0.001

^2^Adjusted *P* value: *<0.05, **<0.01, ***<0.001

**Table S2:** Organisms used for core-genes analysis.

| **^1^Assembly accession** | **^2^Organism** |
| --- | --- |
| GCF_000317695.1 | *Anabaena cylindrica* PCC 7122 |
| GCF_000312705.1 | *Anabaena* sp. 90 |
| GCF_001277295.1 | *Anabaena* sp. WA102 |
| GCF_009498015.1 | *Anabaena* sp. YBS01 |
| GCF_015245355.1 | *Anabaenopsis elenkinii* CCIBt3563 |
| GCF_002368175.1 | *Calothrix* sp. NIES-2098 |
| GCF_019977735.1 | *Calothrix* sp. PCC 7716 |
| GCF_003367075.2 | *Cylindrospermopsis raciborskii* Cr2010 |
| GCF_021650815.1 | *Cylindrospermopsis raciborskii* KLL07 |
| GCF_001548455.1 | *Fischerella* sp. NIES-3754 |
| GCF_022376295.1 | *Nodularia sphaerocarpa* UHCC 0038 |
| GCF_003054475.1 | *Nodularia spumigena* UHCC 0039 |
| GCF_000196515.1 | *Nostoc azollae* 0708 |
| GCA_000009705.1 | *Nostoc* sp. PCC 7120 |
| GCF_000020025.1 | *Nostoc punctiforme* PCC 73102 |
| GCF_009873495.1 | *Nostoc* sp. ATCC 53789 |
| GCF_019598945.1 | *Sphaerospermopsis torques-reginae* ITEP-024 |
| GCF_009856605.1 | *Trichormus variabilis* 0441 |
| GCF_000204075.1 | *Trichormus variabilis* ATCC 29413 |

**Table S6:** *C. raciborskii* strains resistant to phages

| **Strain** | **Ancestor** | **Isolating phage** | **Method** | **Resistant** |
| --- | --- | --- | --- | --- |
| RND1 | WT-D | Cr-LKS5 | L | Yes |
| RND2 | WT-D | Cr-LKS4 | L | Yes |
| RND3 | WT-D | Cr-LKS6 | L | Yes |
| RND4 | WT-D | Cr-LKS5 | S | Yes |
| RNE1 | WT-E | Cr-LKS6 | L | Yes |
| RNE2 | WT-E | Cr-LKS4 | L | Yes |
| RNE3 | WT-E | Cr-LKS5 | S | Yes |
| RNF1 | WT-F | Cr-LKS4 | L | Yes |
| RNF2 | WT-F | Cr-LKS5 | L | Yes |
| RNF3 | WT-F | Cr-LKS5 | L | Yes |
| RNF4 | WT-F | Cr-LKS5 | L | Yes |

**Table S7:** Number of glycosyltransferase genes in cyanobacteria strains.

| **Genome Name** | **GT** | **Morphology** |
| --- | --- | --- |
| *Prochlorococcus marinus* NATL2A | 14 | unicellular |
| *Prochlorococcus marinus* MIT9215 | 16 | unicellular |
| *Synechococcus* sp. WH 8109 | 17 | unicellular |
| *Prochlorococcus marinus* pastoris CCMP 1986 | 18 | unicellular |
| *Prochlorococcus marinus* MIT9313 | 18 | unicellular |
| *Synechococcus* sp. CC9605 | 21 | unicellular |
| *Gloeobacter violaceus* PCC 7421 | 22 | unicellular |
| *Cyanobium* sp. NIES-981 | 23 | unicellular |
| *Synechococcus* sp. NIES-970 | 23 | unicellular |
| *Synechococcus elongatus* UTEX 2973 | 25 | unicellular |
| *Synechococcus elongatus* PCC 7942 | 25 | unicellular |
| *Synechococcus* sp. RCC307 | 25 | unicellular |
| *Gloeobacter violaceus* PCC 7421 | 28 | unicellular |
| *Synechococcus* sp. LTW-R | 31 | unicellular |
| *Synechococcus* sp. PROS-7-1 | 33 | unicellular |
| *Thermosynechococcus* sp. B0 | 33 | unicellular |
| *Thermosynechococcus elongatus* PKUAC-SCTE542 | 36 | unicellular |
| *Euhalothece natronophila* Z-M001 | 37 | unicellular |
| *Cyanobium gracile* PCC 6307 | 42 | unicellular |
| *Synechocystis* sp. PCC 6714 | 44 | unicellular |
| *Synechocystis* sp. PCC 6803 | 49 | unicellular |
| *Microcystis panniformis* FACHB-1757 | 51 | unicellular |
| *Halothece* sp. PCC 7418 | 53 | unicellular |
| *Geminocystis* sp. NIES-3708 | 56 | unicellular |
| *Microcystis aeruginosa* NIES-298 | 59 | unicellular |
| *Rippkaea orientalis* PCC 8801 | 62 | unicellular |
| *Crocosphaera subtropica* BH68 | 63 | unicellular |
| *Microcystis viridis* NIES-102 | 63 | unicellular |
| *Acaryochloris marina* MBIC11017 | 69 | unicellular |
| *Cyanothece* sp. PCC 7425 | 79 | unicellular |
| *Gloeothece verrucosa* PCC 7822 | 80 | unicellular |
| *Gloeocapsa* sp. PCC 7428 | 75 | unicellular |
| *Cylindrospermopsis curvispora* GIHE-G1 | 33 | filamentous |
| *Moorena producens* PAL 15AUG08-1 | 35 | filamentous |
| *Crinalium epipsammum* PCC 9333 | 40 | filamentous |
| *Cylindrospermopsis raciborskii* N8 | 40 | filamentous |
| *Cylindrospermopsis raciborskii* Cr2010 | 42 | filamentous |
| *Pseudanabaena cinerea* FACHB-1277 | 48 | filamentous |
| *Trichodesmium erythraeum* IMS101 | 49 | filamentous |
| *Anabaenopsis elenkinii* CCIBt3563 | 51 | filamentous |
| *Arthrospira platensis* NIES-39 | 51 | filamentous |

**Table S7 continued:**

| **Genome Name** | **GT** | **Morphology** |
| --- | --- | --- |
| *Trichormus azollae* 0708 | 52 | filamentous |
| *Anabaena* sp. WA102 | 52 | filamentous |
| *Limnospira indica* PCC 8005 | 53 | filamentous |
| *Geitlerinema* sp. PCC 7407 | 53 | filamentous |
| *Anabaena* sp. 90 | 54 | filamentous |
| *Dolichospermum* sp. UHCC 0315A | 54 | filamentous |
| *Nodularia spumigena* CCY9414 | 55 | filamentous |
| *Phormidium yuhuli* AB48 | 58 | filamentous |
| *Planktothrix agardhii* NIES-204 | 61 | filamentous |
| *Fischerella* sp. JS2 | 62 | filamentous |
| *Spirulina major* PCC 6313 | 62 | filamentous |
| *Oscillatoria acuminata* PCC 6304 | 64 | filamentous |
| *Halomicronema hongdechloris* C2206 | 65 | filamentous |
| *Calothrix parietina* PCC 6303 | 66 | filamentous |
| *Anabaena cylindrica* PCC 7122 | 68 | filamentous |
| *Planktothrix rubescens* 7821 | 69 | filamentous |
| *Anabaenopsis circularis* NIES-21 | 70 | filamentous |
| *Dolichospermum flos-aquae* CCAP 1403/13F | 70 | filamentous |
| *Oscillatoria nigro-viridis* PCC 7112 | 75 | filamentous |
| *Nostoc sphaeroides* Kutzing En | 75 | filamentous |
| *Cylindrospermum stagnale* PCC 7417 | 77 | filamentous |
| *Planktothricoides raciborskii* FACHB-1370 | 77 | filamentous |
| *Leptodesmis sichuanensis* PKUAC-SCTA121 | 80 | filamentous |
| *Fischerella* sp. NIES-4106 | 83 | filamentous |
| *Calothrix* sp. NIES-2098 | 83 | filamentous |
| *Trichormus variabilis* NIES-23 | 85 | filamentous |
| *Cylindrospermum* sp. NIES-4074 | 86 | filamentous |
| *Nostoc punctiforme* PCC 73102 | 86 | filamentous |
| *Nostoc linckia* NIES-25 | 88 | filamentous |
| *Nostoc* sp. PCC 7120 | 92 | filamentous |
| *Nodularia sphaerocarpa* UHCC 0038 | 94 | filamentous |
| *Leptolyngbya boryana* IAM M-101 | 97 | filamentous |
| *Nostoc carneum* NIES-2107 | 97 | filamentous |
| *Trichocoleus desertorum* NBK24 | 97 | filamentous |
| *Kovacikia* sp. CCNU0001 | 99 | filamentous |
| *Microcoleus* sp. PCC 7113 | 106 | filamentous |
| *Calothrix* sp. PCC 7507 | 113 | filamentous |
| *Leptolyngbya* sp. NIES-3755 | 118 | filamentous |

**Table S8:** Mutations evolved in *Nostoc* 7120 strains under nitrogen starvation with no phage selection.

| **Strain** | **Chromosome** | **Position in the chromosome** | **Mutation** | **Annotation** | **Locus tag** | **Functional annotation** |
| --- | --- | --- | --- | --- | --- | --- |
| F2N | BA000019 | 1,467,025 | G→T | V430F (GTC→TTC) | *alr1236* → | hypothetical protein |
| F1N | BA000019 | 1,546,431 | (A)_7→8_ | coding (156/1722 nt) | *all1304* ← | sulfate permease family protein |
| F2N | BA000019 | 3,953,357 | (T)_6→7_ | coding (313/819 nt) | *all3278* ← | L,D-transpeptidase-like protein |
| F1N | BA000019 | 4,575,663 | A→G | V222V (GTT→GTC) | *all3786* ← | hypothetical protein |
| F1N | BA000019 | 4,575,708 | A→G | C207C (TGT→TGC) | *all3786* ← | hypothetical protein |
| BN | BA000019 | 4,722,133 | ins of GGT | coding (989/1152 nt) | *all3916 ←* | FecR-like protein |
| F1N | BA000019 | 6,038,590 | T→C | V96A (GTG→GCG) | *alr5066* → | UDP-N-acetylmuramate dehydrogenase |
| F2N | BA000019 | 6,286,722 | G→A | V318I (GTT→ATT) | *alr5269* → | glycolate dehydrogenase FAD-linked subunit |

**Supplementary figures:**


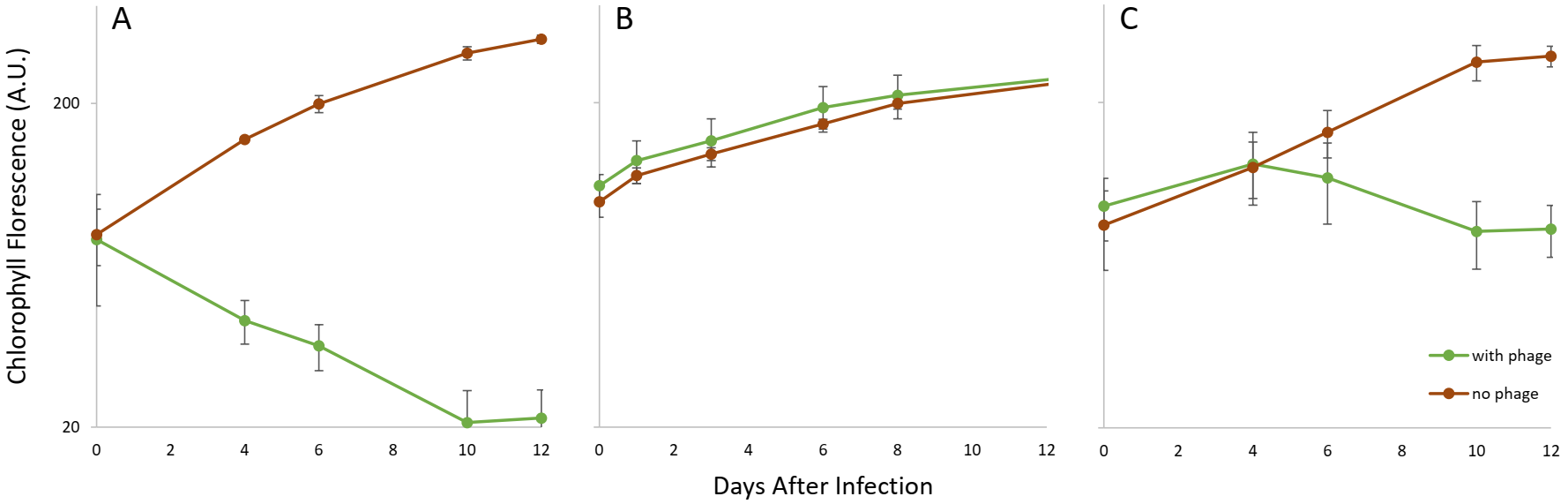
**Figure S1:** **Susceptibility tests of *Nostoc* Resistant strains**. Population dynamics of a susceptible strain (WT-D; A), a fully resistant strain (RND2; B), and a partially resistant strain (RND5; C) when infected with A-4L (light green) or not infected (brown) over 12 days post inoculation. Cyanobacteria growth was estimated using chlorophyll auto -fluorescence as a proxy for biomass. A.U., arbitrary units. Average and standard deviation of 3 biological replicates.


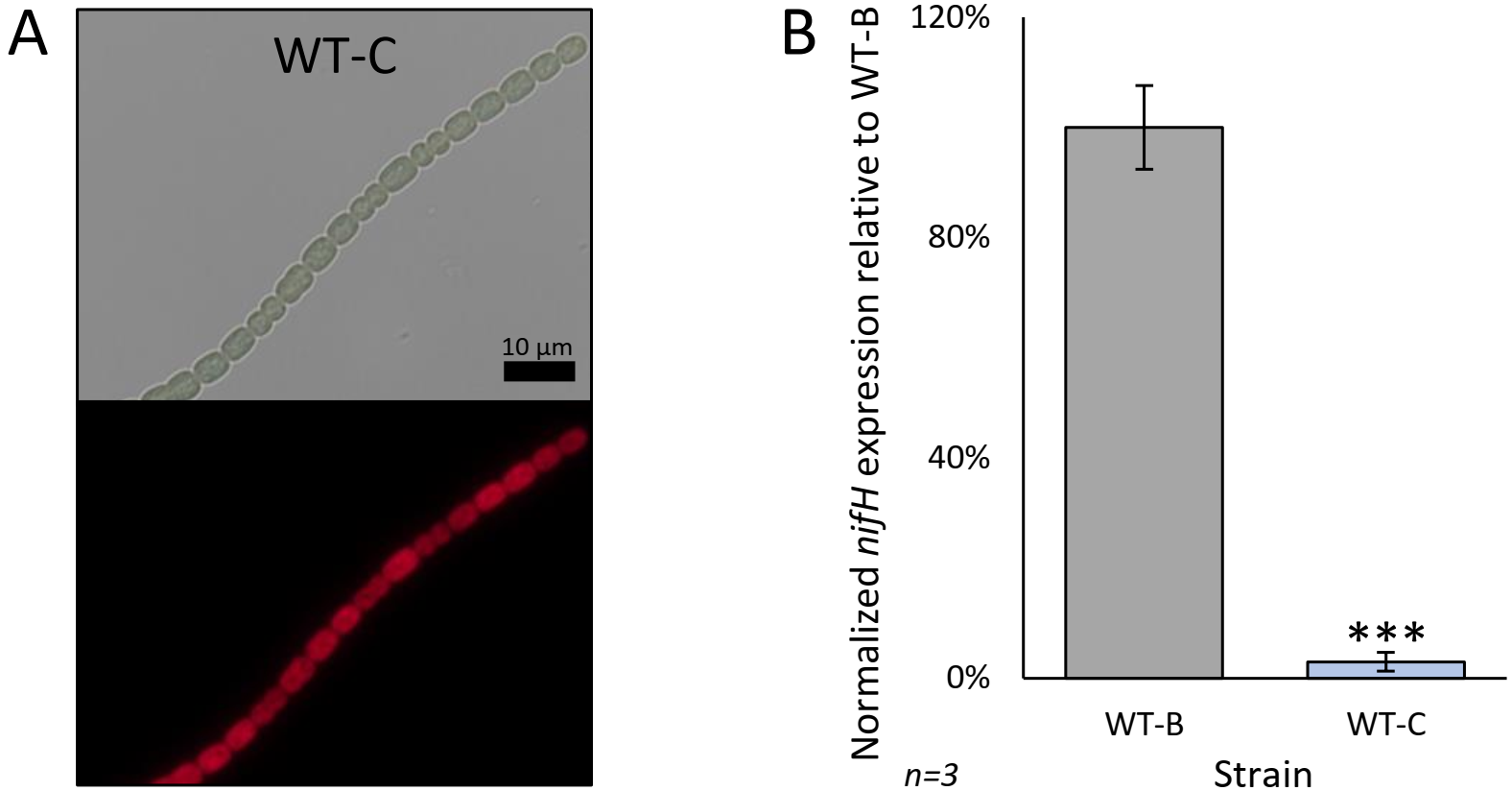


**Figure S2**: **Morphology and physiology of** **WT-C. A**, Bright field and the corresponding fluorescence images of WT-C (*Nostoc* 7120) 48 hours after fixed nitrogen stepdown. Both uncappable producing heterocysts, Scale equals 10 µm. **B**, Expression of *nifH* gene in WT-C (light blue) relative to WT-B (grey). The transcript levels of *nifH* values are normalized to the transcript levels of *rnpB*. Data shown are average and standard deviation of n biological replicates. ***, p < 0.001.

**Supplementary references:**

1. Kolan D, Cattan-Tsaushu E, Enav H, Freiman Z, Malinsky-Rushansky N, Ninio S, et al. Tradeoffs between phage resistance and nitrogen fixation drive the evolution of genes essential for cyanobacterial heterocyst functionality. *ISME J* 2024; **18**: wrad008.

2. Khudyakov IY, Golden JW. Different functions of HetR, a master regulator of heterocyst differentiation in *Anabaena* sp. PCC 7120, can be separated by mutation. *Proc Natl Acad Sci U S A* 2004; **101**: 16040–16045.

3. Nayar AS, Yamaura H, Rajagopalan R, Risser DD, Callahan SM. FraG is necessary for filament integrity and heterocyst maturation in the cyanobacterium *Anabaena* sp. strain PCC 7120. *Microbiology* 2007; **153**: 601–607.

4. Flores E, Pernil R, Muro-Pastor AM, Mariscal V, Maldener I, Lechno-Yossef S, et al. Septum-localized protein required for filament integrity and diazotrophy in the heterocyst-forming cyanobacterium *Anabaena* sp. strain PCC 7120. *J Bacteriol* 2007; **189**: 3884–3890.

5. Cannon RE, Shane MS, Whitaker JM. Interaction of *Plectonema borianum* (cyanophyceae) and the LPP-cyanophages in continuous culture. *J Phycol* 1976; **12**: 418–421.

6. Schwartz DA, Lindell D. Genetic hurdles limit the arms race between *Prochlorococcus* and the T7-like podoviruses infecting them. *ISME J* 2017; **11**: 1836–1851.
